# AlN MEMS filters with extremely high bandwidth widening capability

**DOI:** 10.1038/s41378-020-00183-5

**Published:** 2020-09-07

**Authors:** Anming Gao, Kangfu Liu, Junrui Liang, Tao Wu

**Affiliations:** 1 Department of Electrical and Computer Engineering, University of Illinois at Urbana-Champaign, Urbana, IL 61820 USA; 2 School of Information Science and Technology (SIST), ShanghaiTech University, Shanghai, 201210 China; 3 University of Chinese Academy of Sciences, No. 19(A) Yuquan Road, Shijingshan District, Beijing, China

**Keywords:** NEMS, Electrical and electronic engineering

## Abstract

This paper presents radio frequency (RF) microelectromechanical system (MEMS) filters with extremely high bandwidth widening capability. The proposed filtering topologies include hybrid configurations consisting of piezoelectric MEMS resonators and surface-mounted lumped elements. The MEMS resonators set the center frequency and provide electromechanical coupling to construct the filters, while the lumped-element-based matching networks help widen the bandwidth (BW) and enhance the out-of-band rejection. Aluminum nitride (AlN) S0 Lamb wave resonators are then applied to the proposed filtering topologies. AlN S0 first- and second-order wideband filters are studied and have shown prominent performance. Finally, the AlN S0 first-order wideband filter is experimentally implemented and characterized. The demonstrated first-order filter shows a large fractional bandwidth (FBW) of 5.6% (achieved with a resonator coupling of 0.94%) and a low insertion loss (IL) of 1.84 dB. The extracted bandwidth widening factor (BWF) is 6, which is approximately 12 times higher than those of the current ladder or lattice filtering topologies. This impressive bandwidth widening capability holds great potential for satisfying the stringent BW requirements of bands n77, n78, and n79 of 5G new radio (NR) and will overcome an outstanding technology hurdle in placing 5G NR into the marketplace.

## Communication: high-performance radio frequency MEMS filters

Researchers in China have developed a new type of radio frequency filter with extremely bandwidth widening capabilities, which will be useful for 5G networks. The approach is a hybrid configuration combining a microelectromechanical system (MEMS) filters combined with lumped elements. Dr. Tao Wu’s team at ShanghaiTech University began by modeling MEMS filters based on aluminum nitride resonators. They found that the first-order filter of proposed topology was several times better than existing systems at bandwidth, and that second-order filters improved rejection even further. Next, they fabricated a filter based on this design and characterized it. The bandwidth was approximately 12 times better than conventional standalone resonators. These designs will be valuable to filter out unwanted signals in 5G communications technologies.

## Introduction

The proliferation of 5G communications has led to the allocation of wider bands for faster data transmission and processing^1^. Radio frequency (RF) bandpass filters, which are used to select the desired signals while suppressing the unwanted signals, are the keys to define these frequency bands. Therefore, the realization of filters with a wide bandwidth (BW) is attracting tremendous attention. Filters based on various technologies, including microstrip, metal cavity, dielectric, lumped element, and acoustic or microelectromechanical systems (MEMS), have all been developed in recent years. Among these technologies, MEMS filters stand out for their compact size, low cost, and excellent filtering performance (especially the sharp roll-off). These advantages make MEMS filters the most promising solution to 5G filtering applications. Researchers have made extensive investigations of MEMS filtering technologies in three main directions: exploiting resonant modes with higher coupling (*k*_t_^2^)^2–4^, exploring strong piezoelectricity materials (e.g., AlScN^5–7^, LiNbO_3_^1^), and hybridizing acoustic resonators to expand the bandwidth^8–10^. For example, the shear horizontal (SH0) mode and first-order asymmetric (A1) mode in lithium niobate (LiNbO_3_) have been demonstrated with high *k*_t_^2^ values of 50% and 22%, respectively^11–13^. Acoustic resonators with these modes can be used to construct wideband filters^14,15^. However, the severe power-handling issue and temperature instability make the two modes still not promising enough for wideband or high-frequency applications. Nevertheless, AlN is the first choice in piezoelectric resonators^16–18^ and sensors^19^ since it has a high power-handling capability and excellent thermal stability and offers a mature process that can be integrated with complementary metal-oxide-semiconductor (CMOS) integrated circuits (ICs)^20^. Scandium (Sc) has been studied for its doping with AlN to increase the piezoelectric coefficients that determine the couplings of the resonators^21,22^. It has been reported that doped Sc_0.12_Al_0.88_N can increase the piezoelectric coefficient *d*_33_ by 50% and reduce the stiffness constant *c*_33_ by 10%^23^. Thanks to this achievement, *k*_t_^2^ is improved by 1.7 times. Nevertheless, the doped AlN always comes with the degradation of the quality factor, which directly affects the insertion loss (IL) of the filters. In addition to the two developments above, researchers have also made efforts to hybridize filters using quartz surface acoustic wave (SAW) resonators and lumped elements or microwave transmission lines^8–10^. However, these hybridized filters suffer from small bandwidth (BW), poor roll-off, and low out-of-band rejection.

To solve the above issues, this paper proposes AlN MEMS filters with extremely high bandwidth widening capabilities. The wideband filters utilize AlN Lamb wave resonators in conjunction with surface-mounted lumped elements. Compared with conventional ladder and lattice topologies, the proposed filters show a much larger bandwidth widening capability.

This paper is organized as follows: the Method and Modeling section presents the models of the two filtering topologies: first-order and second-order wideband filters. Both filters are designed based on a general MEMS resonator. The Material and Device section illustrates the AlN MEMS first-order and second-order wideband filters. It begins with the introduction of the AlN S0 Lamb wave resonator, followed by the implementation of the two wideband filtering topologies. The Results and Discussion section shows the experimental realization of the AlN first-order wideband filter. The filter fabrication, assembly, and characterization results are covered. The final section concludes this paper with some potential future endeavors.

## Method and modeling

### First-order wideband filter

**Fig. 1:**
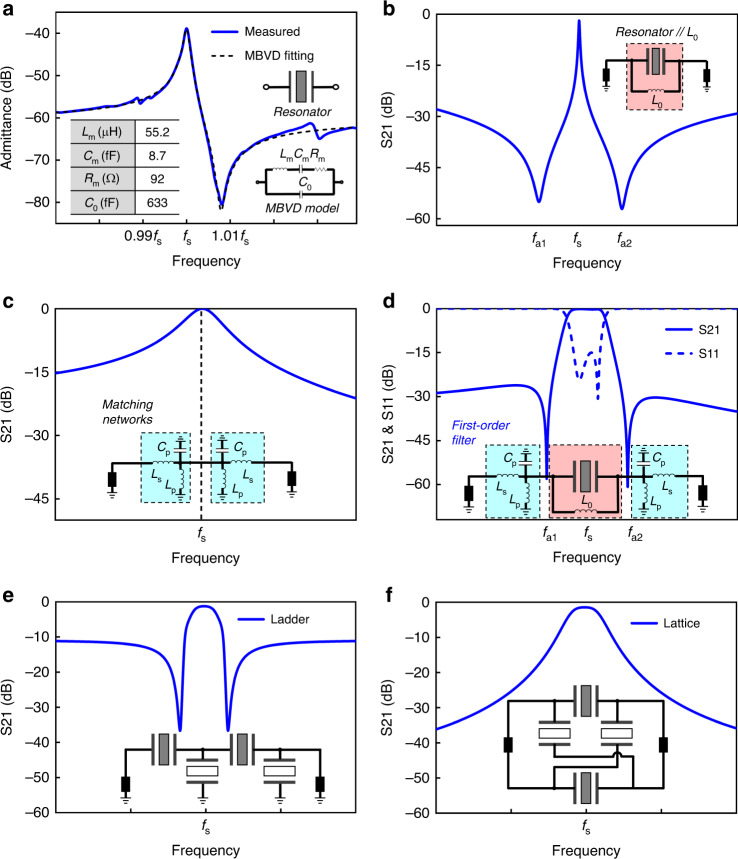
The topology and principle of the proposed first-order filter. **a** MEMS resonator representation and its MBVD fitting model. The admittance response is fitted with MBVD parameters. **b** The MEMS resonator and the paralleled inductor $L_0$. Two symmetric transmission zeros are generated. The center frequency is the same as the resonant frequency ($f_s$) of the MEMS resonator. **c** Two three-lumped-element matching networks. Each consists of a series inductor, a shunt inductor, and a shunt capacitor. The two matching networks behave like a wide bandpass filter centered at $f_s$. **d** Proposed first-order wideband filter. It is constructed from the combination of the topologies in (**b**) and (**c**). The filter is centered at $f_s$. It features a large bandwidth and two deep TZs. **e** The frequency response and topology of a ladder-type filter. **f** The frequency response and topology of a lattice-type filter

The filter construction starts with the MEMS resonator. The electrical response of a MEMS resonator is often represented with the modified Butterworth–Van Dyke (MBVD) model, in which *C*_0_ is the static capacitance of the resonator and *L*_m_, *C*_m_, and *R*_m_ are the motional inductance, motional capacitance, and motional resistance, respectively. Extraction of the MBVD parameters can be found in many literature reports^24–29^. The motional branch forms the mechanical resonance equivalently in the electrical domain via piezoelectricity. As an example, the measured response of a MEMS resonator is plotted and fitted in Fig. 1a. The demonstrated resonator has a typical resonant frequency *f*_s_ and an antiresonant frequency *f*_p_. The quality factor and the piezoelectric coupling factor are two important parameters for the performance of the resonators, which can be related to the MBVD model parameters. Therefore, the MBVD model is extracted from the measured response and will be used as the basis for the following filter analysis.

As shown in Fig. 1d, the proposed first-order filter is obtained from the combination of the topology in Fig. 1b, c, which consists of a MEMS resonator, a parallel inductor *L*_0_, and a pair of three-lumped-element matching networks, which includes a series inductor *L*_s_, a shunt inductor *L*_p_, and a shunt capacitor *C*_p_. When the MEMS resonator parallel with the inductor *L*_0_, two symmetric transmission zeros *f*_a1_ and *f*_a2_ are generated, as indicated in Fig. 1b. The filter is centered at the same resonant frequency (*f*_s_) as the MEMS resonator, and it features a large bandwidth and two deep transmission zeros (TZs).

The series resonance frequency *f*_s_ is the frequency where *L*_m_ and *C*_m_ cancel each other out, while the antiresonance frequency *f*_p_ arises when *C*_0_ and *C*_m_ collectively cancel out *L*_m_. The parallel *L*_0_ is added to decouple the antiresonance and generate two symmetric TZs: *f*_a1_ and *f*_a2_. The two TZs are determined by the MBVD model and *L*_0_, as shown in Fig. 1b and computed as

1 $$f_{{\rm{a}}1,{\rm{a}}2} = \frac{1}{{2\pi }}\sqrt {\frac{{\omega _{\rm{s}}^2} \mp {\sqrt {t_1t_2} } + {\omega _{{\rm{s}}0}^2} + {C_{\rm{m}}L_0\omega _{\rm{s}}^2\omega _{{\rm{s}}0}^2}}{2}}$$

where

$$t_1 = C_{\rm{m}}L_0\omega _{\rm{s}}^2\omega _{{\rm{s}}0}^2 + \omega _{\rm{s}}^2 - 2\omega _{\rm{s}}\omega _{{\rm{s}}0} + \omega _{\rm{s}}^2$$

$$t_2 = C_{\rm{m}}L_0\omega _{\rm{s}}^2\omega _{{\rm{s}}0}^2 + \omega _{\rm{s}}^2 + 2\omega _{\rm{s}}\omega _{{\rm{s}}0} + \omega _{\rm{s}}^2$$

$$\omega _{\rm{s}} = 1/\sqrt {C_{\rm{m}}L_{\rm{m}}}$$

$$\omega _{{\mathrm{{{s}}}}0} = 1/\sqrt {C_0L_0}$$

If we set, *ω*_s_ = *ω*_*s*0_, then

2 $$L_0 = \frac{{L_{\rm{m}}C_{\rm{m}}}}{{C_0}} = \frac{1}{{\omega _{\rm{s}}^2C_0}} = \frac{1}{{(2\pi f_{\rm{s}})^2C_0}}$$

Then, Eq. (1) can be rewritten as

3 $$f_{{\rm{a}}1,{\rm{a}}2} = \frac{{\omega _{\rm{s}}}}{{2\pi }}\sqrt {\frac{{2 + C_{\rm{m}}/C_0 \pm \sqrt {(2 + C_{\rm{m}}/C_0)^2 - 4} }}{2}}$$

Since the piezoelectric coupling factor of the resonator device is proportional to *C*_m_/*C*_0_^3^ and the TZs determine the maximum bandwidth of the filter, Eq. (3) implies that a higher bandwidth widening capability can be obtained by using resonators with a larger piezoelectric coupling factor.

As demonstrated in Fig. 1c, the lumped-element matching network consists of a series inductor *L*_s_, a shunt inductor *L*_p_, and a shunt capacitor *C*_p_. The purpose of the matching networks is to widen the bandwidth of the filter. The three-lumped elements resonate at *f*_s_ and are regulated by

4 $$\left( {\frac{1}{{L_{\rm{p}}}} + \frac{1}{{L_{\rm{s}}}}} \right) \cdot \frac{1}{{C_{\rm{p}}}} = \omega _{\rm{s}}^2$$

Only when *L*_s_, *L*_p_, and *C*_p_ obey Eq. (4) can we obtain a functional matching network. If any two of three are determined, then the third one can be acquired by Eq. (4). Therefore, only two parameters are independent, and the left parameter is dependent. In later discussion, *L*_s_ and *L*_p_ will be chosen as independent variables and *C*_p_ as the dependent variable.

**Fig. 2:**
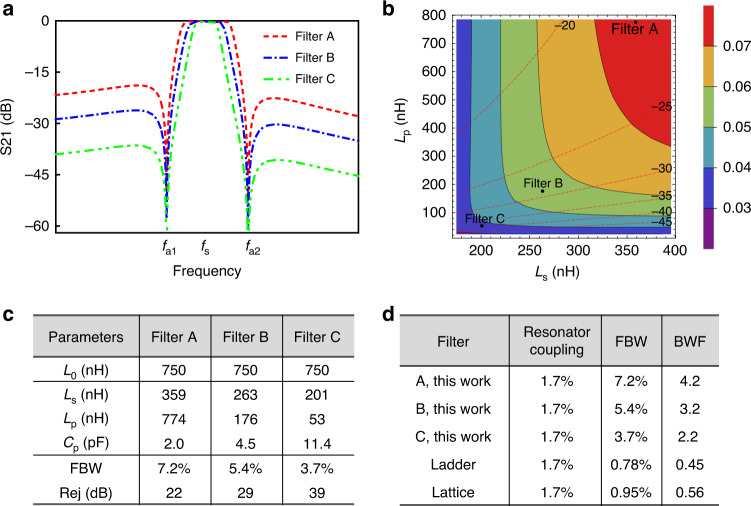
Simulated performance proposed first-order filter. **a** Simulated filtering performance of three first-order filters with different matching networks or matching lumped elements. Filter A has the largest *L*_s_ and *L*_p_, Filter B has an intermediate *L*_s_ and *L*_p_, and Filter C has the smallest *L*_s_ and *L*_p_. **b** Simulated filtering FBW performance versus different *L*_s_ and *L*_p_; the colored solid contour lines are the boundaries for the FBW performance of the filter topology, while the dashed lines are boundaries for out-of-band rejection performance. **c** Parameters and comparison of the three first-order filters. **d** Comparison of the bandwidth widening effect of three first-order filters and conventional ladder and lattice filtering topologies

Finally, as shown in Fig. 1d, the proposed filter is formed by combining the two topologies in Fig. 1b, c. In this filtering topology, the MEMS resonator and *L*_0_ provide sharp roll-off, TZs, and set the center frequency of the filter, while the matching network, which in essence is an LC bandpass filter, offers the wide bandwidth for the filter. The filtering topology enjoys high-performance flexibility by tuning the lumped elements in the matching networks. As shown in Fig. 2b, when *L*_s_ and *L*_p_ are given a different value while *C*_p_ is determined by Eq. (4) *L*_s_ and *L*_p_ have different effects on the filter bandwidth and out-of-band rejection. In general, larger *L*_s_ and *L*_p_ lead to a greater fractional bandwidth (FBW), while *L*_p_ has a greater impact on out-of-band rejection than *L*_s_, and a smaller *L*_p_ corresponds to greater out-of-band rejection. *Q*_s_ of the resonator and the lumped elements together determine the IL of the filter. By leveraging *L*_s_ and *L*_p_, it is possible to achieve wide bandwidth and excellent out-of-band rejection simultaneously.

As an example of the tunability, Fig. 2a demonstrates three filters (named Filters A, B, and C) with different matching networks (for quantification purposes, we assume *f*_s_ = 229 MHz and *f*_p _ = 231 MHz based on a measured MEMS resonator; *k*_t_^2^ is then computed to be 1.7%). The values of the three-lumped elements *L*_s_, *L*_p_, and *C*_p_ are listed in Fig. 2c. Among the three filters, Filter A has the largest *L*_s_ and largest *L*_p_. *C*_p_ is therefore the smallest. As explained above, a larger *L*_s_ and *L*_p_ produce a wider BW, and a larger *L*_p_ leads to smaller out-of-band rejection. Therefore, Filter A is assumed to have the largest BW and the smallest out-of-band rejection, which is verified by the simulation in Fig. 2a. Filter C has the smallest *L*_s_ and *L*_p_, so it has the smallest BW and the largest out-of-band rejection. The comparison of the performance of the three filters is concluded in Fig. 2c. Figure 2b illustrates the simulated filtering FBW performance versus different *L*_s_ and *L*_p_ values; the dashed lines show the out-of-band rejection values for guidance.

The proposed first-order filters stand out for their wide FBWs. To justify their bandwidth widening capabilities, the three filters (A, B, and C) are compared with existing widely used ladder and lattice filtering topologies. The bandwidth widening factor (BWF), defined as the quotient of the FBW and the resonator’s electromechanical coupling, is adopted for evaluation. Conventional ladder and lattice topologies^27^, which are shown in Fig. 1e, f, typically achieve FBWs of 1/3–1/2 of the resonator’s coupling^27,28^. Taking the assumed MEMS resonator that has an electromechanical coupling of 1.7%, for example, the FBWs of the ladder and lattice topologies are 0.78% and 0.95%, respectively. Figure 2d compares the BWFs of the three first-order filters and the ladder and lattice topologies. Apparently, the proposed first-order filters have the largest BWF of 4.2, which is 9.1 and 7.5 times higher than the ladder and lattice topologies, respectively.

Though showing a large FBW, the roll-off and out-of-band rejection of the first-order wideband filter still needs enhancement. For further improvement, a second-order wideband filter is then proposed.

### Second-order wideband filter

**Fig. 3:**
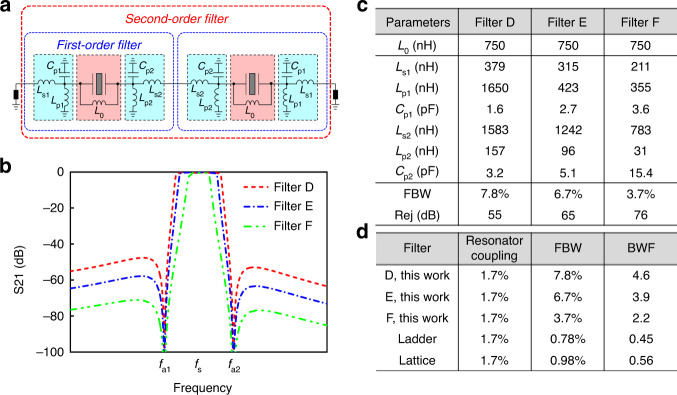
The topology and performance of the proposed second-order filter. **a** Proposed second-order wideband filter and its simulated filtering response. **b** Simulated filtering performance of the three second-order wideband filters with different matching networks or matching lumped elements. Filter D has the largest *L*_s_, *L*_s2_, *L*_p_ and *L*_p2_, Filter E has an intermediate *L*_s_, *L*_s2_, *L*_p_ and *L*_p2_, and Filter F has the smallest *L*_s_, *L*_s2_, *L*_p_ and *L*_p2_. **c** Comparison of the three second-order wideband filters. **d** Comparison of the bandwidth widening effect of three second-order wideband filters and conventional ladder and lattice filtering topologies

As shown in Fig. 3a, the second-order wideband filter is composed of two series of first-order wideband filters. The two first-order wideband filters are designed to be image symmetrical for simultaneous matching of the two ports. In order to have more freedom of matching impedance, one side of the matching network is changed to be *L*_s2_, *L*_p2_, and *C*_p2_. The roles of *L*_0_, *L*_s1_, *L*_p1_, *C*_p1_, *L*_s2_, *L*_p2_, and *C*_p2_ are the same as those described in the first-order wideband filter, and they are also regulated by Eqs. (2) and (4). The second-order wideband filter features a much better performance of out-of-band rejection and roll-off. In addition, the FBW has also been improved. The simulated response of the second-order wideband filter in Fig. 3b demonstrates a large FBW of 6.7% and an extremely high out-of-band rejection of over 60 dB. Moreover, the roll-off is also significantly enhanced, and the -30dB shape factor is only 1.05.

The second-order wideband filter enjoys favorable performance flexibility since it has four tuning elements (*L*_s1_, *L*_p1_, *L*_s2_, and *L*_p2_), while the first-order filter has only two tuning elements (*L*_s_ and *L*_p_). Similarly, to demonstrate the tuning capability, another three second-order wideband filters (Filters D, E, and F) are constructed using different matching networks. The values of the lumped elements of each filter are listed in Fig. 3c. The functions of *L*_s1_, *L*_s2_, *L*_p1_, and *L*_p2_ are similar to those in the first-order case. Filter D has the largest *L*_s1_, *L*_s2_, *L*_p1_, and *L*_p2_, so it has the widest FBW but the smallest out-of-band rejection (Fig. 3b). Filter F has the smallest *L*_s1_, *L*_s2_, *L*_p1_, and *L*_p2_, which corresponds to the smallest FBW but the highest out-of-band rejection. Filter E has intermediate values of *L*_s1_, *L*_s2_, *L*_p1_, and *L*_p2_, and its FBW and out-of-band rejection are between Filter Ds and Filter Fs. Overall, the second-order filters exhibit much better out-of-band rejection and roll-off than the first-order filters.

The bandwidth widening effect of the second-order wideband filters is also researched and compared in Fig. 3d. Filter D has the highest BWF of 4.6 because it uses the largest *L*_s1_, *L*_s2_, *L*_p1_, and *L*_p2_. This BWF could be further increased if higher *L*_s1_, *L*_s2_, *L*_p1_, and *L*_p2_ were applied. Of course, the compromise will be the degradation of out-of-band rejection. Filter F has the smallest BWF of 2.2, but its out-of-band rejection is as high as 76 dB. Compared with the first-order wideband filter C, which has the same BWF of 2.2, the second-order wideband filters achieve much better out-of-band rejection performance.

The MEMS resonators in the proposed first-order and second-order filters are constructed by the MBVD model. There are no specific requirements of the types of resonators. The MEMS resonators could be various MEMS resonators, such as AlN Lamb wave resonators, SAW resonators, FBARs, or LiNbO_3_ A1 resonators. To validate the feasibility and achieve multiple-frequency filters, the following section will replace the general MEMS resonator with a concrete AlN Lamb wave resonator.

## Material and device design

### AlN Lamb wave resonator

AlN has emerged as the most suitable material for the transduction of acoustic waves because of its excellent performance and manufacturability. Engineered AlN has shown desirable properties of large phase velocity, high thermal conductivity, low acoustic loss, and relatively small temperature coefficients of frequency (TCFs)^19^, which make it preferable for resonator applications. AlN S0 Lamb wave resonators have attained great success in terms of high resonant frequency, great power-handling capability, high *Q*, and low-frequency drift^30–32^. Most importantly, Lamb wave resonators make it possible to integrate multiple-frequency resonators^26^ or filters^33^ on piezoelectric films of the same thickness. However, the relatively low electromechanical coupling (< 2%) of the AlN S0 Lamb wave resonators limits their use in RF wideband filters. As analyzed, the proposed wideband filtering topologies can significantly extend the FBWs, so in this section, AlN MEMS wideband filters will be designed and implemented.

**Fig. 4:**
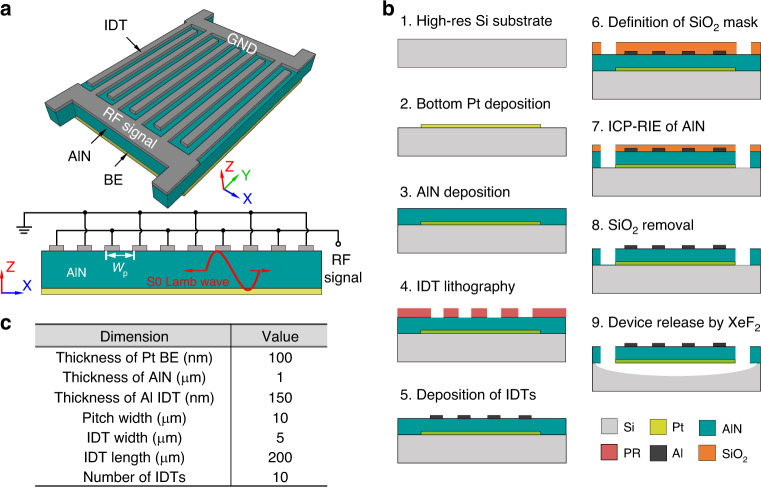
Fabrication process of the AlN Lamb wave resonator. **a** Prospective view and cross-sectional view of the AlN Lamb wave resonator. The resonator consists of three layers: bottom electrode, AlN thin film, and top IDTs. The Lamb wave is excited in the stack. **b** Fabrication process of the AlN S0 Lamb wave resonator. **c** Dimensions of the designed AlN S0 Lamb wave resonator

As illustrated in Fig. 4a, the designed AlN S0 Lamb wave resonator is comprised of top interdigitated transducers (IDTs), a suspended AlN thin film, and a bottom electrode (BE). The top IDTs are alternatingly connected to the ground and RF signal, and the BE is electrically floating. The resonator is designed with four tethers at the four corners to increase the structure robustness and power-handling capability. The resonant frequency of the intended S0 mode is designed to be approximately 450 MHz. The BE and top IDTs are chosen to be 100 nm Pt and 150 nm Al, respectively. The middle AlN thin film is approximately 1 μm thick. The pitch width of the IDTs is 10 μm, which forms the designed resonant frequency of approximately 450 MHz. The parameters of the AlN S0 Lamb wave resonator are described in Fig. 4c.

Figure 4b shows the fabrication process of the AlN S0 resonator. It starts with a high-resistivity Si wafer. Pt (100 nm) is deposited by evaporation and then lifted off with negative photoresist AZ5214E. Then, 1 μm thick AlN is reactively sputtered, followed by IDT lithography with photoresist SPR220. The 150 nm Al is sputtered and lifted off as the top IDTs. A hard mask of SiO_2_ is chosen to define the AlN film by inductively coupled plasma-reactive ion etching (ICP-RIE). After the ICP-RIE, the remaining SiO_2_ is removed in HF.

**Fig. 5:**
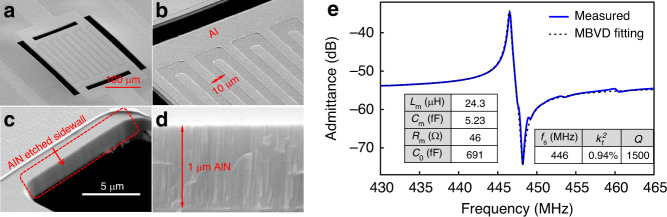
SEM image and measured response of fabricated AlN S0 Lamb wave resonator. SEM images of the (**a**) fabricated AlN S0 Lamb wave resonator, (**b**) Al IDTs, (**c**) etched AlN sidewall, and (**d**) 1μm AlN crystal. **e** Measured admittance response of the fabricated AlN S0 Lamb wave resonator. The measured response is fitted by the MBVD model

Finally, the resonator is exposed to XeF_2_ for release. Figure 5a–d shows the scanning electron microscope (SEM) images of the fabricated device and its zoomed-in structures.

The fabricated resonator was measured in dry air with an Agilent PNA-L 5230A at room temperature. The measured admittance result is shown in Fig. 5e. The resonator has a measured resonant frequency at 446 MHz, an electromechanical coupling of 0.94% and a *Q* of 1500. The resonator’s measured response is then fitted by the MBVD model. The fitted MBVD *L*_m_, *C*_m_, *R*_m_*C*_0_ parameters are listed in the inset of Fig. 5e and will be used in the AlN S0 first- and second-order hybrid wideband filters.

### AlN S0 first-order wideband filter

**Fig. 6:**
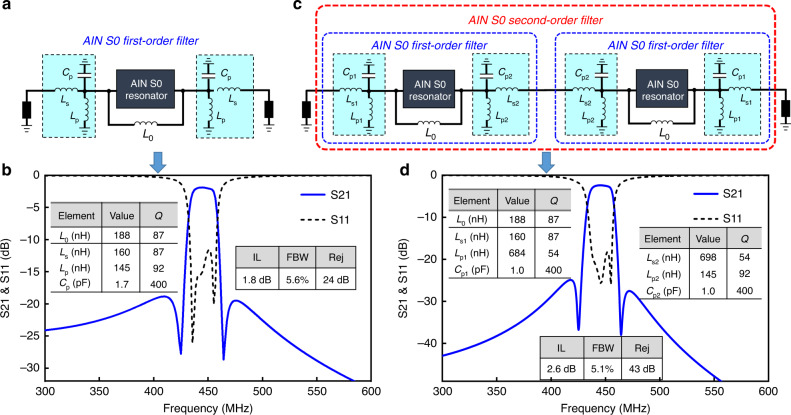
The topology and performance of the proposed AlN S0 first-order and second-order filter. **a** Topology of the AlN S0 first-order filter. **b** Simulation of the AlN S0 first-order filter. The lumped element parameters and the extracted filtering performance is added in the inset. **c** Topology of the AlN S0 second-order wideband filter. **d** Simulation of the AlN S0 second-order wideband filter. The lumped element parameters and the extracted filtering performance are added in the inset

The principle of the first-order wideband filter has been introduced in the Method and Modeling section. In this section, we will replace the general MEMS resonator in Fig. 1d with the fabricated AlN S0 Lamb wave resonator, as indicated in Fig. 6a. The AlN S0 Lamb wave resonator is modeled by using the MBVD circuit lumped elements listed in the inset of Fig. 5e. According to Eq. (2), the parallel *L*_0_ is calculated to be 188 nH. For the other three-lumped elements, *L*_s_ and *L*_p_ are chosen to be 160 nH and 145 nH, respectively, and *C*_p_ is computed to be 1.7 pF (shown in the inset of Fig. 6b). Generally, inductors and capacitors are lossy due to their finite *Q*s. According to the datasheets of current lumped-element vendors, the *Q*s of *L*_0_, *L*_s_, *L*_p_, and *C*_p_ are assumed to be 87, 87, 92, and 400, respectively.

The simulation result of the AlN S0 first-order wideband filter is illustrated in Fig. 6b. The filter is centered at 446 MHz with a low IL of 1.8 dB, a high FBW of 5.6%, and an out-of-band rejection of 24 dB. As noted, the electromechanical coupling of the AlN S0 resonator is only 0.94%. However, it reveals a very high BWF of 6 after implementing the first-order filter topology.

The out-of-band rejection of the AlN S0 first-order wideband filter is moderate. For improvement, an AlN S0 second-order wideband filter is then evaluated below.

### AlN S0 second-order wideband filter

The topology of the AlN S0 second-order filter is similar to that of the second-order filter in Fig. 3a. Shown in Fig. 6c, the AlN S0 second-order wideband filter consists of two series of AlN S0 first-order filters. *L*_0_ is the same as that in the AlN S0 first-order filter. The lumped elements (*L*_s1_, *L*_p1_, *C*_p1_, *L*_s2_, *L*_p2_, and *C*_p2_) are adjusted accordingly for better impedance matching. Figure 6d shows the simulated performance of the AlN S0 second-order filter. The values of the lumped elements used in the filter are provided in the inset of Fig. 6d. The second-order filter is also centered at 446 MHz. It has an IL of 2.6 dB, a wide FBW of 5.1%, and very high out-of-band rejection (e.g., 43 dB at 300 MHz). The 2.6 dB IL is higher than the 1.8 dB in the AlN S0 first-order filter because of the additional cascaded loss from the lumped elements. This 2.6 dB IL can be reduced if high-*Q* lumped elements are available or at higher frequencies where high-*Q* lumped elements are attainable. In short, the second-order filters have much higher out-of-band rejection than the first-order filters, and the payoff is the complexity of the circuit as well as the cost of extra lumped components.

## Results and discussion

### Implementation of the AlN MEMS filter

Due to the resource limitation and the similarity among the proposed filters, we choose only the AlN S0 first-order wideband filter to implement. The dimensions of the fabricated AlN S0 first-order wideband filter are listed in Fig. 4c. The filter is implemented by first fabricating the AlN S0 Lamb wave resonator and then integrating it with the matching networks on a printed circuit board (PCB). The fabrication of the AlN S0 resonator utilizes a process described in detail in Fig. 4b. The AlN S0 resonators are fabricated on a resonator chip. It is then cut into smaller pieces for later PCB mounting.

**Fig. 7:**
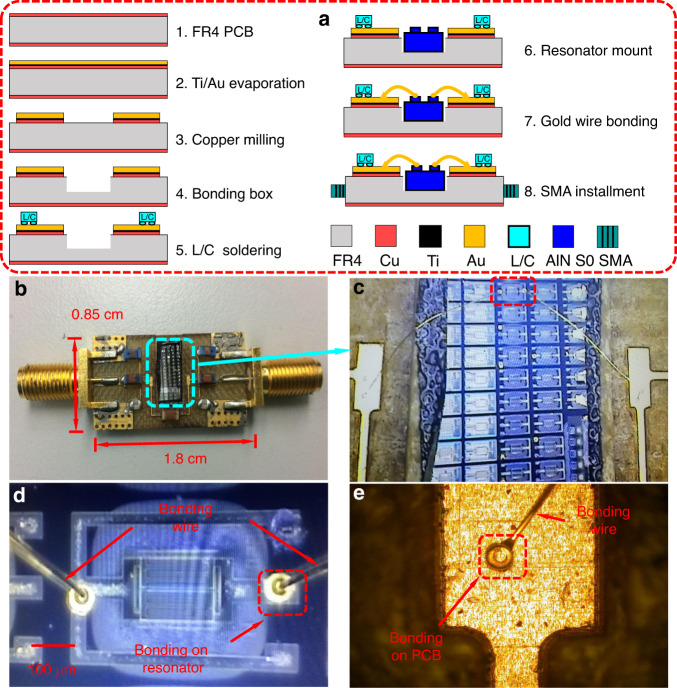
Assembly process of the proposed AlN S0 first-order filter. **a** Assembly process of the AlN S0 first-order wideband filter. **b** Fabricated AlN S0 first-order wideband filter. **c** Mounted AlN resonator chip. **d** Magnified view of the bonded AlN. **e** Bonded wire on the PCB

As shown in Fig. 7a, the filter assembly process starts with PCB preparation, which includes size planning and surface cleaning. The chosen PCB is based on an FR4 with a thickness of 1.524 mm, a dielectric constant (*ε*_*r*_) of 4.8, a dielectric loss tangent tan (*δ*_D_) of 0.008, and a copper cladding thickness of 18 μm. Integration generally requires wire bonding (step 7) at 120 °C. However, the copper cladding would be oxidized very quickly and make the bonding fail. To avoid this, 50 nm platinum (Pt) and 250 nm gold (Au) are continuously evaporated on the PCB. The Pt works as an adhesion layer, while the Au layer has the two-fold purpose of antioxidation and easy bonding (a gold substrate usually provides the best bonding ease). Subsequently, the copper is milled to form the signal traces and bonding pads. For easy bonding, a bonding box with a depth of 600 nm is opened in the center of the PCB. The bounding box helps the top surfaces of the resonator chip and the PCB stay on the same plane. It also ensures that the bonding wires do not touch the corner of the chip. The lumped elements of the surface mount inductors and capacitors are then soldered on the PCB with a hot air gun and soldering paste. The resonator chip is attached to the bonding box and then wire bonded to the signal traces on the PCB. Finally, two SMA connectors are installed for measurement.

The PCB assembly has considerable influence on the filter. There are several aspects we need to monitor so that minimal PCB influence is introduced. First, the parasitic effect from the PCB should be reduced. In our circuit model in Fig. 6a, the filter contains the lumped elements and AlN MEMS resonators only. There is no parasitic content in the filter circuit. However, in the real case, when we assemble the filter to the PCB, the parasitic effect always exists and influences the filter performance. For low-frequency filters, the parasitic effect is not that significant and can be compensated by tuning the three-lumped elements (tuning *C*_p_ would be a good option). For high-frequency filters, the parasitic effect becomes dominant, which requires an electromagnetic environment to be applied to the filter circuit. Second, the grounding pads on the PCB are key. Grounding is critical because it generates different levels of parasitic capacitance. To achieve a good PCB assembly, the grounding pads on the PCB must be carefully designed so that minimal parasitic capacitance is generated. Third, the bonding wires introduce parasitics. Bonding wires can be thought of as a kind of inductor, which can also exert an influence on the filter. One has to plan the assembly beforehand so that the least length of bonding wire is needed.

Figure 7 demonstrates the assembly process and images of the fabricated filter with some integration details. The fabricated filter (Fig. 7b) has dimensions of 1.85 cm × 0.85 cm. This dimension is smaller than that of most microstrip filters but still larger than those of pure acoustic filters (SAW or BAW filters). The main causes of the relatively large size are die-level bonding and manual mounting, which require additional operation space. By using a more compact flip-chip approach that involves chip-scale lumped elements, the form factor of the filter can be substantially improved. Figure 7c shows the AlN resonator chip featuring arrays of resonators. The target AlN S0 resonator is at the top center as circled. Figure 7d is the magnified view of the bonded AlN S0 resonator. The two bonding wires connect the two ports (input and output ports) of the resonator. The two wires start from the resonator and end on the bonding pads on the PCB, as shown in Fig. 7e.

### Characterization results

**Fig. 8:**
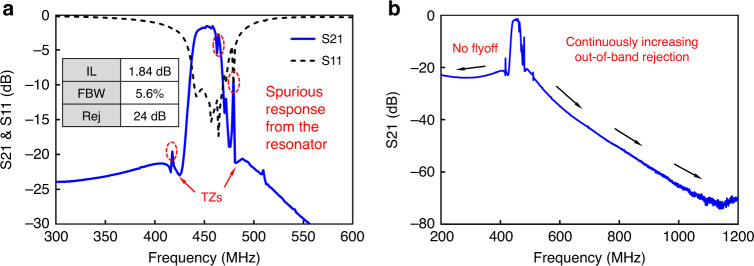
Measured response of the fabricated AlN S0 first-order filter. **a** Measured response (S21 and S11) of the fabricated AlN S0 first-order filter from 300 MHz to 600 MHz. **b** Measured response (S21) of the same filter from 200 to 1200MHz

The fabricated AlN S0 first-order wideband filter is measured by an Agilent PNA-L 5230 A analyzer in dry air and at room temperature. The measurement results are shown in Fig. 8a. The filter demonstrates a low IL of 1.84 dB, a wide FBW of 5.6%, and out-of-band rejection of 24 dB. Overall, the measurement agrees well with the simulation results shown in Fig. 6b. As noted, there are two mismatches: the depth of the two TZs and the spurious response. The measurement has shallower TZs than the simulation. This can be attributed to the fact that the actual *Q* of *L*_0_ is lower than the simulated *Q* provided by the vendors. Generally, a higher *Q*_L0_ gives a deeper TZ depth. The spurious response in the measurement comes from the spurious modes of the resonator. The simulation adopts the MBVD model, which does not incorporate spurious modes. This explains why we see the spurious response in the measurement only. Techniques have been reported to suppress the spurious modes in AlN Lamb wave resonators^34–37^. By applying these spurious mode suppression techniques, the spurious response of the filter could be eradicated.

Figure 8b plots the measured response of the filter over a wider frequency range between 200 and 1200 MHz. As seen, the out-of-band rejection on the left has no fly off. This advantage overcomes the long-existing fly off issues in microstrip filters or purely acoustic filters. In addition, the out-of-band rejection on the right continuously increases up to 75 dB. Additionally, there are no spurious harmonic responses. This merit overtakes its counterparts of microstrip filters and purely acoustic filters.

Finally, it is worthwhile to notice the bandwidth widening capability of the achieved filter. The coupling of the AlN S0 Lamb wave resonator is 0.94%, and the achieved FBW of the filter is 5.6%. Therefore, the BWF is calculated to be 6.0, which is approximately 12 times higher than that of the conventional ladder or lattice topologies. As predicted from Eq. (3), a higher FBW can be achieved by implementing this bandwidth widening technique on resonators with a higher piezoelectric coupling factor.

## Conclusion

Wideband MEMS filters have been analyzed, designed, fabricated and characterized in this work. Two filtering topologies (first- and second-order) are first proposed and researched. Then, AlN S0 Lamb wave resonators are applied to the filtering topologies and show excellent performance of low IL, wide bandwidth, and high out-of-band rejection. To validate the simulated filters, the AlN S0 first-order filter is chosen for implementation, which progresses from the resonator fabrication to the final filter assembly. The demonstrated AlN S0 first-order filter has a high FBW of 5.6%, a low IL of 1.84 dB, and an out-of-band rejection larger than 24 dB. Considering the low coupling (0.94%) of the AlN S0 resonator, the BWF is as high as 6, which is approximately 12 times higher than that of the ladder or lattice topologies. Furthermore, the demonstrated AlN S0 first-order filter features far-frequency suppression without spurious responses. The presented hybrid filters hold great potential for various filtering applications, especially for the current 5G NR bands of n77, n78, and n79.

Moreover, very recently, high-frequency on-chip rolled-up inductors with miniaturized sizes have been reported^38,39^. The self-rolled-up inductor can achieve a maximum *Q* factor of over 12 at 3.5 GHz and 10 nH inductance with a footprint area of only 15 × 19 μm^2^, which is 0.1% of that of planar spiral inductors^38^. Their fabrication process could be incorporated with that of the Lamb wave resonators. In addition, it has been shown that piezoelectric resonators can be integrated with capacitors on the same membrane^40^, especially on AlN^41^, since the AlN thin film sputtering and resonator device fabrication process is compatible with CMOS ICs. Therefore, it is possible to achieve the inductances *L*_0_, *L*_s_, and *L*_p_ with these rolled-up inductors and the capacitance *C*_p_ with the AlN Lamb wave resonators and finally attain multiple-frequency on-chip hybrid filters. This will be our future development.
